# Brassinosteroid Signaling in Plant–Microbe Interactions

**DOI:** 10.3390/ijms19124091

**Published:** 2018-12-17

**Authors:** Mei-Hui Yu, Zhe-Ze Zhao, Jun-Xian He

**Affiliations:** School of Life Sciences and State Key Laboratory of Agrobiotechnology, The Chinese University of Hong Kong, Shatin, New Territories, Hong Kong, China; cketnn7@hotmail.com (M.-H.Y.); zheze.zhao@hotmail.com (Z.-Z.Z.)

**Keywords:** brassinosteroids (BRs), plant–microbe interactions, defense responses, plant innate immunity, signal transduction

## Abstract

As sessile organisms, plants are frequently exposed to different stress conditions caused by either biotic or abiotic factors. Understanding the mechanisms that underlie plant interaction with the biotic and abiotic environments is fundamental to both plant biotechnology and sustainable agriculture. Brassinosteroids (BRs) are a group of plant-specific steroidal compounds essential for normal growth and development. Recent research evidence indicates that BRs are also actively involved in plant–environment interactions and play important roles in shaping plant fitness and the growth–defense trade-offs. In this minireview, we focus our attention on recent advances in the understanding of BR functions in modulating plant interactions with different pathogenic microbes, with particular focus on how BR signaling primes the plant innate immunity pathways and achieves a trade-off between growth and immunity.

## 1. Classification of Plant Pathogens

Living in natural environments, plants are subject to constant attack by various microbial pathogens and insect herbivores, and diseases caused by microbial pathogens are the major threats to plant growth and agricultural productivity. Depending on whether or not they cause disease, phytopathogens can be divided into avirulent pathogens and virulent pathogens. Avirulent pathogens carry dominant avirulence genes that can be recognized by dominant resistance (*R*) genes carried by plant hosts, leading to resistance in the hosts. This is called “gene-for-gene” recognition or resistance. When lacking either the avirulence gene in the pathogen and/or the *R* gene in the host, the host becomes susceptible and the pathogen is virulent [1].

According to their lifestyles or way of deriving nutrients, plant pathogens are divided into three groups: biotrophs, necrotrophs, and hemibiotrophs. Biotrophs, including the phytopathogenic viruses and subsets of bacteria and fungi, gain nutrients from living host cells without killing them. They infect and colonize young plant cells with active metabolism and usually have a long symbiotic phase with the host cells [2]. Necrotrophs, including a large number of bacteria, fungi, and oomycete species, promote the destruction of host cells and derive nutrients from the dead or dying cells [3], and therefore are more adapted to the metabolism of older plants and/or their senescing parts with active catabolic pathways [2]. The third group, hemibiotrophs, which include some bacteria and many fungi, initially have a biotrophic stage in the early infection process, but later become necrotrophic. However, the duration of the biotrophic or necrotrophic phase varies significantly among different hemibiotrophic pathogens [1,3,4].

Different pathogens infect plants using different strategies. Biotrophic pathogens enter the plant surface through wounds and stomata and later multiply in the intercellular spaces. They infect plant cells through developing haustoria, the specialized feeding structure, to slowly drain plant resources and gradually decrease plant fitness, but do not kill the cells [3]. The hemibiotrophic bacteria, such as *Pseudomonas syringae* pv. *tomato* DC3000, do not cause host cell death in the early stages of infection, but in the later stages of infection, induce chlorosis and necrosis in host tissue by producing toxins. *P. syringae* also inject effector proteins into host cells through a type III secretion system, suppressing host immunity and causing disease [1,5]. Necrotrophic pathogens, such as the fungi *Botrytis cinerea* and *Alternaria brassicicola*, penetrate the plant surface through small wounds or cracks in the cuticle or enter through the stomata. They can kill host cells at very early stages of infection by secreting cell wall-degrading enzymes and other lytic enzymes to cause tissue damage. They also produce phytotoxic compounds such as phytotoxins and proteins to promote host cell death [1,3]. 

After being attacked by different pathogens, plants express a complex set of responses to defend themselves. The common responses include the production of antimicrobial metabolites such as phytoalexins, cell wall fortification through the production of callose and lignin, the rapid production of reactive oxygen species (ROS) called oxidative burst, and programmed cell death known as the hypersensitive response (HR). These “basal defenses” can provide effective resistance to biotrophic pathogens, but not to necrotrophic ones [1]. For necrotrophic pathogens that can overcome a plant’s basal defenses, additional defense mechanisms are therefore required, such as specific responses to pathogen-derived toxins or damage-associated molecular patterns, induction of systematic resistance, and activation of different hormone signaling [3]. It has been demonstrated that the salicylic acid (SA)-dependent pathway is mostly involved in defense against biotrophic pathogens, while jasmonic acid (JA) and ethylene (ET) signaling pathways are mainly associated with defense against necrotrophic pathogens, although extensive crosstalk exists between these hormones [1,4]. 

## 2. Plant Innate Immunity and BR Signal Transduction Pathways

**Figure 1:**
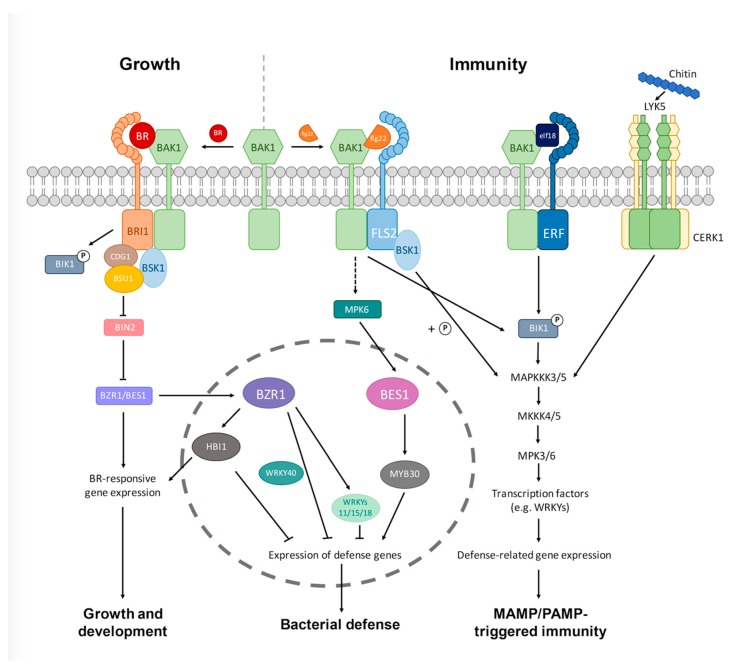
A simplified model for BR interaction with the innate immunity pathways in plants. The BR signaling pathway (far left) has crosstalk with different MTI or PTI pathways at multiple levels (receptor, cytoplasmic, or transcriptional). The crosstalk is either BAK1-dependent (flg22- or elf18-triggered immunity) or independent (chitin-triggered immunity), and MTI/PTI triggered by different MAMPs/PAMPs converge on similar downstream signaling events, including the formation of MAPK cascades, activation of transcription factors, and defense-related gene expression, among others. Please note that the two RLCKs, BIK1 and BSK1, directly connect the FLS2-BAK1 and/or ERF-BAK1 receptor complex to the downstream MAPK components, and that the two homologous transcription factors BZR1 and BES1 play distinct roles in flg22-triggered bacteria defense. BZR1 inhibits plant immunity by suppressing the defense-related gene expression, whereas BES1 enhances plant immunity by promoting MYB30-mediated signaling pathways. Whether BZR1 and BES1 play different roles in regulating the trade-offs between growth and immunity under different environmental conditions needs further studies to elucidate. CERK1, chitin-elicitor receptor kinase 1; FLS2, FLAGELLIN SENSITIVE 2; ERF, elongation factor-TU (EF-Tu) RECEPTOR; LYK5, lysin motif receptor kinase 5; MTI: MAMP-triggered immunity; PTI: PAMP-triggered immunity; MAMP: microbe-associated molecular patterns; PAMP: pathogen-associated molecular patterns; MAPK: mitogen-activated protein kinase; RLCK: receptor-like cytoplasmic kinase.

To fight against pathogen attacks, plants have evolved a multilayered self-protection system. In addition to the physical and chemical barriers mentioned above, the primary or core layer of this protection system is the innate immunity that is activated upon pathogen attacks [6]. Plant innate immunity is triggered by the perception of the conserved molecular signatures of many pathogens, named microbe- or pathogen-associated molecular patterns (MAMPs or PAMPs) by pattern-recognition receptors (PRRs) at the cell surface. Well-characterized PAMPs include flg22, a 22-amino acid (aa) peptide derived from bacterial flagellin; elf18, an 18-aa peptide from the elongation factor Tu (EF-Tu), and chitin from fungal cell walls, which are detected by FLAGELLIN SENSITIVE 2 (FLS2); EF-Tu RECEPTOR (ERF), and lysin-motif (LysM) containing proteins, respectively [7,8]. Recognition of MAMPs or PAMPs activates PRRs and initiates a downstream signaling cascade conferring resistance to a broad range of pathogens. The whole process is called MAMP/PAMP-triggered immunity (MTI or PTI) [7,8,9] (Figure 1). Some pathogens can inject specific effector proteins into plant cells and induce effector-triggered immunity (ETI), which is initiated by the recognition of effectors by disease-resistance proteins encoded by the *R* genes [7,10]. ETI is genetically similar to PTI and both induce a suite of defense responses including a reactive oxygen burst, increased expression of pathogen-response genes, and mitogen-activated protein kinase (MAPK) signaling. However, ETI is quicker, stronger, and often induces the hypersensitive response (HR) that causes localized cell death to prevent pathogens from spreading further and accessing water and nutrients [1,11].

Both PTI and ETI are modulated by plant hormones, and the three stress hormones of salicylic acid (SA), jasmonic acid (JA), and ethylene (ET) are the primary signals [6]. However, recent studies have indicated that the growth hormones BRs and gibberellic acid (GA) also play important roles [12,13]. BRs and GA undergo crosstalk with plant defense signaling pathways to fine-tune the trade-offs between growth and immunity under different physiological conditions [12,13,14]. 

BRs were originally identified as a group of growth-promoting hormones [15], but later were found to be critical for many other steps in plant development, including seed germination, vegetative and reproductive development, senescence, and responses to different stresses [16,17,18,19]. To date, a main signal transduction pathway of BRs has been established in the model plant *Arabidopsis thaliana*, which has paved the way for further understanding the molecular mechanisms of BRs in regulating different plant processes. According to the current model, BR signals are perceived by the BR receptor BR-INSENSITIVE 1 (BRI1) and its coreceptor BRI1-ASSOCIATED KINASE 1 (BAK1), both of which are plasma membrane-localized leucine-rich repeat receptor-like kinases (LRR-RLKs) [20,21,22,23]. Upon BR binding, the BRI1 kinase is activated by autophosphorylation and BAK1 transphosphorylation and dissociated from the negative regulators BR KINASE INHIBITOR 1 (BKI1) [24] and BOTRYTIS-INDUCED KINASE 1 (BIK1) [25]. The activated BRI1 then sequentially phosphorylates and activates the downstream components BR SIGNALING KINASE 1 (BSK1) [26,27], CONSTITUTIVE DIFFRENTIAL GROWTH 1 (CDG1) [28], and the phosphatase BRI1-SUPPRESSOR 1 (BSU1), which leads to the dephosphorylation and inactivation of the GSK3/Shaggy-like kinase BR-INSENSITIVE 2 (BIN2) that negatively regulates BR signaling [28,29]. In the absence of BR or when the BR level is low, BIN2 activity is high and it will target the two master transcription factors BRASSINAZOLE-RESISTANT 1 (BRZ1) and BRI1-EMS-SUPPRESSOR 1 (BES1) (also known as BZR2) through phosphorylation and inhibit their function by inducing phosphorylation-triggered protein degradation [30,31,32,33], reduced DNA binding activity [34], and cytoplasmic retention [35,36]. When the BR level is high, BIN2 is inactivated and its inhibition of BZR1 and BES1/BZR2 is released by triggering their dephosphorylation and translocation to the nucleus, where they bind BR-responsive gene promoters, inducing transcriptional reprogramming and as such, shaping various BR-signaling outputs [37,38,39,40]. A simplified model of BR signal transduction pathways in *Arabidopsis* is shown in Figure 1. 

## 3. Functions of BRs in Different Plant–Pathogen Interactions

**Figure 2:**
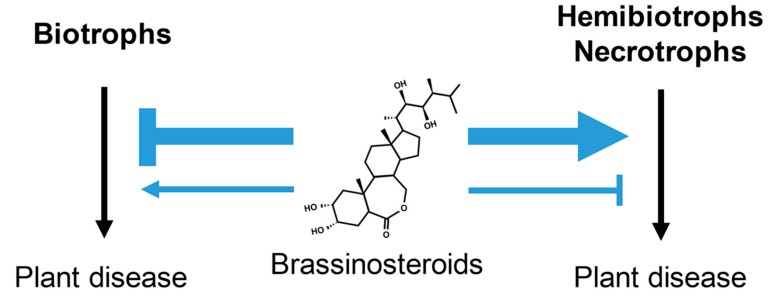
Brassinosteroids (BRs) modulate plant interactions with all three types of trophic pathogens; however, they appear to induce resistance to most biotrophs, but susceptibility to most necrotrophs and hemibiotrophs. The black arrows indicate induction of plant disease by different types of pathogens. The blue arrows and T-shaped lines signify promoting and inhibitory effects of BRs on pathogen-induced disease, respectively. The thickness of each arrow or line is proportional to the strength of BRs in the denoted action.

BRs have been implicated in plant interactions with all three trophic-type pathogens (Figure 2), but their effects on them (inducing either defense or susceptibility) appear to depend not only on the pathogen’s lifestyle, but infection strategy and also on how BRs are administrated to the plants (exogenously or endogenously) [4]. Accordingly, the roles of BRs in plant defense will be discussed in the context of interactions with different types of pathogens using evidence from both BR-treatment experiments and studies using different BR mutants.

### 3.1. Roles of BR in Plant Interaction with Biotrophic Pathogens

Biotrophic pathogens live with plant hosts in a “pretended harmony”, and therefore their damage to the plant is relatively mild and not destructive [1]. According to published data, BRs seem to be able to increase resistance and protect plants from majority of these pathogens. For example, early field studies in crop plants suggest that exogenously applied BR could confer tolerance to plants from a wide spectrum of pathogen infections. In potato, BR treatment reduced the damage to plants caused by phytophthora, a genus of oomycetes (water molds) [41], and a similar effect of BR was also observed in tomato, cucumber, sugar beet, and some other plants [42]. In tobacco, pretreatment of plants with brassinolide (BL), the most active BR, gave rise to increased resistance to the biotrophic bacterial pathogen *Pseudomonas syringae* pv. *tabaci* (*Pst*) and the biotrophic fungus *Oidium* sp. (powdery mildew) [43]. In rice plants, BR is able to enhance resistance to the fungal pathogen *Magnaporthe grisea* and the bacterial pathogen *Xanthomonas oryzae* pv*. oryzae* [43]. The effect of BR was found to be not only local, but also systemic. However, BR-induced systemic resistance appears to be independent of the systemic acquired resistance (SAR) induced by necrotizing pathogens or SAR inducers such as SA. Therefore, the authors proposed the possible existence of a steroid hormone-mediated disease resistance (BDR) in BR-treated plants, at least in tobacco [43]. However, the detailed mechanisms of BDR remain to be clarified.

Phytopathogenic viruses invade the plant surface and propagate in their cytoplasm as biotrophic pathogens, although some of them can cause very serious disease [2]. BR has been shown to play diverse roles in coping with viral infections, mediating either defense or susceptibility. Early studies have shown that BR can induce resistance to the viral pathogen tobacco mosaic virus (TMV) in tobacco and rice. BL treatment enhanced the N-gene-mediated resistance in response to necrotic-type infection with TMV, resulting in smaller size of lesions and restricted spread of the virus in the infection site [43]. Recent studies confirmed that BL treatment can increase systemic resistance to TMV through the production of ROS in *Nicotiana benthamiana* and that TMV resistance is impaired when the BR receptor NbBRI1 is silenced [44]. BR-induced inhibition of TMV replication involves the accumulation of H_2_O_2_ and nitric oxide (NO) synthesis, which are required for the upregulation of defense-associated gene expression. BR treatment was also shown to provide tolerance to *Arabidopsis* plants to cucumber mosaic virus (CMV) infection, thus BR signaling was necessary for CMV resistance [45]. BR-induced CMV tolerance was associated with the antioxidant system by boosting antioxidative enzymes’ activities, such as superoxide dismutase (SOD), peroxidase (POD), catalase (CAT), and ascorbate peroxidase (APX). Consistently, the antioxidative enzymes’ activities were elevated in the positive BR signaling mutant *bes1-D* during CMV infection [45]. 

However, the effects of BR in plant viral defense are not always positive; there is also evidence showing negative roles of BR. For example, BR was recently reported to increase the susceptibility of rice plants to rice black-streaked dwarf virus (RBSDV) infection, and the increased susceptibility was attributed to BR suppression of JA-mediated defense responses [46]. In addition, the expression of BR biosynthetic genes (*OsCPDs* and *OsDWF4*) and signaling genes (*OsBRI1* and *OsBZR1*) was downregulated, whereas that of JA biosynthetic genes was upregulated when the rice plants were exposed to RBSDV [46], suggesting an antagonistic relationship between BR and JA effects in viral defense. 

### 3.2. Roles of BR in Plant Interaction with Hemibiotrophic and Necrotrophic Pathogens

Many studies have been conducted on BR effects on plant interaction with hemibiotrophic and necrotrophic pathogens, possibly due to the large populations and wide host spectra of these pathogens. The effects of BRs with these pathogens appear to be pleiotropic; they either promote resistance, increase susceptibility, or have no effect, depending on the pathogens and plant species involved. For instance, exogenously applied BR was reported to induce resistance in barley plants to several fungal pathogens exhibiting different trophic lifestyles [47]. In particular, application of the epibrassinolide (epiBL) to heads of ‘Lux’ barley reduced the severity of *Fusarium* head blight (FHB) caused by *Fusarium culmorum* by 86% and reduced the FHB-caused loss of grain yield by 33%. In addition, the growth of plants in soil amended with epiBL resulted in 28% and 35% reductions in *Fusarium* seedling blight (FSB) symptoms on the Lux and ‘Akashinriki’ barley, respectively. Transcriptional profiling of these plants during the early stages of FSB development indicated that the expression of genes involved in chromatin remodeling, hormonal signaling, photosynthesis, and pathogenesis were activated as a result of growth in epiBL-amended soil [47]. However, exogenously applied BR showed no effect on inducing the resistance of wild-type *Arabidopsis* plants infected with the hemibiotrophic bacteria *Pseudomonas syringae* pv. *tomato* (*Pto*) DC3000 or the necrotrophic fungus *Alternaria brassicicola* [48]. In rice, instead of enhancing the plant’s resistance, BRs were found to increase the susceptibility to the hemibiotrophic pathogens *Pythium graminicola* and *Meloidogyne graminicola* [49,50]. BR also induced the susceptibility of potato tuber tissues by stimulating the mycelial growth, intensifying the spore formation of *Phytophthora infenstans*, and weakening the immune status of plant tissues [51]. 

The roles of BR have also been demonstrated by using different mutants affected in either BR biosynthesis or signaling. In *Arabidopsis*, for instance, overexpression of *DWARF4*, a gene that encodes for the 22α-hydroxylase that catalyzes a rate-limiting step of BR biosynthesis [52], resulted in enhanced growth but dramatically reduced responses to flg22, a bacterial flagellin epitope [53], suggesting that a proper size of endogenous BR pools is essential for appropriate defense responses. The same authors also demonstrated that ectopic overexpression of BRI1, the BR receptor, dramatically reduced plant responsiveness to flg22 in *Arabidopsis*. However, the *BRI1* hypermorphic allele *BRI1^sud1^* dramatically increased plant responses to flg22 and enhanced plant resistance to *Pto* DC3000, a hemibiotrophic bacterium [53]. Because *BRI1^sud1^* plants have enhanced BR signaling but similar BRI1^sud1^ protein accumulation to wild-type plants, these results suggest that BR signaling functions antagonistically with disease-resistance mechanisms and that balanced BRI1 and BAK1 protein levels are essential for correct MPI signaling [53]. In contrast to BRI1 overexpression, the disruption of BRI1 activity by *BRI1* mutations increases disease resistance to both necrotrophic and hemibiotrophic pathogens, but has no effect on biotrophic pathogens in several plant species including *Brachypodium distachyon* and barley [54,55]. 

BAK1 is a coreceptor of BRI1 in BR signaling and also a coreceptor of the flagellin receptor FLAGELLIN SENSITIVE 2 (FLS2) in PTI signaling [56]. Studies have shown that BAK1-deficient mutants display enhanced susceptibility to infection by necrotrophic fungal pathogens [57,58,59], but increased resistance to biotrophic pathogens [57], suggesting opposing roles of BAK1 in resistance to necrotrophic and biotrophic pathogens. Similar results were observed for the *bes1-D* mutant, which carries a dominant point mutation in the transcription factor BES1 and enhances BR signaling [32]. *bes1-D* is specifically susceptible to *Alternaria brassicicola*, a necrotrophic fungus, but is less susceptible to *Pseudomonas syringae* pv. *tomato* (*Pst*) DC3000, a biotrophic pathogen defined by the authors [60].

### 3.3. Role of BRs in Plant Interaction with Insect Herbivores

Apart from microbial pathogens, plants also face attack from insects. Although defense against insects is not the focus of this review, some recent studies indicate that BRs are also involved in different plant–herbivore interactions; therefore, we will cover this topic briefly here. The herbivore defense system is triggered by the wounded tissues, and the peptide hormone systemin acts as the spreading signal [61]. Previously, the tomato LRR-RK160 (SR160), a BRI1 homologue, was identified as a systemin receptor to activate downstream signaling mechanisms and lead to systemic defense responses after wounding or attack by herbivores [62]. However, subsequent studies could not confirm SR160’s role as a systemin receptor, but suggested that it is only a systemin-binding protein that does not involve in systemin perception or signaling [63,64]. Recently, a distinct LRR-RK, SYR1, was reported to be the genuine systemin receptor. SYR1 can bind systemin with high affinity and specificity in tomato and is important for defense against insect herbivory [65]. Although the systemin receptor was only discovered recently, the PTI-like (PAMP-triggered immunity) responses are believed to occur in plants in response to different HAMPs (herbivore-associated molecular patterns), DAMPs (damage-associated molecular patterns), or effectors [7,66,67]. Perception of these patterns triggers the expression of SA-related defense genes and the concomitant repression of JA-triggered immunity (JATI). 

An earlier work in *Nicotiana attenuata* has revealed that herbivory-induced defense requires NaBAK1, a tobacco homologue of *Arabidopsis* BAK1. NaBAK1 modulates herbivory-induced JA accumulation and the levels of defense-related secondary metabolites [68]. Recent studies show that BRs function in plant defense with insect herbivory through regulating glucosinolate (GS) biosynthesis [69,70]. Preference test and larval feeding experiments using the generalist herbivore, the diamondback moth (*Plutella xylostella*), revealed that the larvae prefer to feed on *Arabidopsis brassinosteroid insensitive 1* (*bri1-5*) plants over wild-type or BRI1-overexpressing transgenic plants [70]. BR was found to inhibit the synthesis of certain GSs [69,70], and this was mediated by a signaling pathway involving the transcription factors BZR1, BES1, and several MYC factors, where BZR1/BES1 inhibited the expression of MYBs and thus resulted in the inhibition of MYB-promoted GS synthesis [69]. BZR1/BIL1 was also reported to increase resistance to thrip feeding, and the enhanced resistance may involve JA signaling [71]. 

## 4. Signaling Mechanisms by Which BRs Modulate Plant–Pathogen Interactions 

In plants, the activation of growth and immunity responses are opposing processes that define a trade-off [14]. Studies in the past few years have suggested that the growth-promoting BRs negatively regulate plant innate immunity (PTI), promoting growth at the expense of defense [14,48,53]. BRs undergo crosstalk with PTI pathways at multiple levels, including the receptor level, cytoplasmic level, and transcriptional level, which involve several BR signaling components (Figure 1). The crosstalk seems to be negative and unidirectional, as the activation of PTI does not affect the analyzed BR signaling steps [13,48,53]. In this section, we will introduce how BR crosstalks with the immune pathways at different signaling stages and ultimately achieves perturbation. We will mainly focus on BR interaction with the flg22-triggered immunity pathway, while other PAMP pathways are touched upon. 

### 4.1. Crosstalk at the Receptor Level

Plants constantly respond and adapt to the changing environment through the surface-localized transmembrane receptor-like kinases (RLKs). RLKs have been reported to control several aspects of plant growth and immune pathways through recognizing pathogen-associated molecular patterns (PAMPs). The *Arabidopsis* leucine-rich repeat RLK (LRR-RLK) BAK1/SERK3 is also suggested as an important trade-off mediator, as it acts as the coreceptor of BRI1 in BR signaling and FLAGELLIN SENSITIVE 2 (FLS2) and ELONGATION FACTOR-THERMO UNSTABLE RECEPTOR (EFR) in plant immune signaling [10]. FLS2 and EFR recognize the PAMPs flagellin and elongation factor Tu (EF-Tu), respectively, and initiate innate immune signaling [7,72,73]. Researches have shown that mutation in BAK1 (*bak1*) results in anomalous flagellin- and EF-Tu-triggered responses, especially in the induction of oxidative burst, suggesting its positive role in innate immune signaling [10]. 

Studies indicate that the BRI1–BAK1 and FLS2–BAK1 complexes coexist in cells, suggesting the potential “rail switch” between BR and PAMP signaling and that BAK1 may be a rate-limiting regulator that acts as a decision node between different pathways [48,74]. Differential phosphorylation by either BRI1 or FLS2 on BAK1 offers cells with dual signaling specificity upon external stimuli [25,75]. Recently, the conserved BAK1 phosphosites that are essential for the immune response but are not required for BAK1-dependent BR-regulated growth have been identified [76] and suggested a phosphocode-dependent dichotomy of BAK1 in regulating growth and the immune response.

Shared as the coreceptor of BRI1 and FLS2, BAK1 is believed to play key roles in mediating the crosstalk between BR and PTI signaling pathways, as BAK1 could become rate-limiting and competed by BRI1 and FLS2 receptors [10]. However, two recent studies provided very different results regarding these possibilities. The study of Albrecht et al. [48] suggested that BAK1 is not a rate-limiting factor in both pathways and that the BR-induced suppression of immune responses via FLS2 was independent of BAK1, because BR treatment did not affect BAK1–FLS2 association in vivo and BR could still inhibit FLS2-mediated MTI responses in the null *bak1-4* mutant. By contrast, the work by Belkhadir et al. [53] demonstrated a different scenario. By using MAMPs that required BAK1 (flg22, elf18, and peptidoglycans) and that did not require BAK1 (chitin), the authors’ results led to the conclusion that BR can inhibit MTI in both BAK1-dependent and -independent manners. In addition, plants overexpressing BRI1 failed to respond to flg22 treatment, similar to *bak1* and *fls2* plants, which also suggested that BR inhibition of MTI requires BAK1. According to Belkhadir et al. [53], BAK1 could become rate-limiting for MTI signaling, as BAK1 was indeed recruited away from MAMP receptors in the BRI1-overexpressing plants.

Apart from BAK1 and BRI1, BR–PTI crosstalk also involves other potential signaling components. BOTRYTIS-INDUCED KINASE 1 (BIK1), a receptor-like cytoplasmic kinase (RLCK), was reported to negatively regulate BR signaling but positively regulate plant immune pathways [73]. BIK1 is a direct substrate of BAK1 and can further associate with the FLS/BAK1 complex for flagellin signaling [73]. Similar to BIK1, BSK1, another RLCK and a BRI1 substrate [26], was also reported to positively regulate plant innate immunity and disease resistance by directly interacting with FLS2 [77]. The *bsk1-1* mutant possessed enhanced susceptibility to a wide range of bacterial, fungal, and oomycetic pathogen species and accumulated low levels of SA after pathogen infection. *bsk1-1* also displayed compromised oxidative burst induced by flg22 [77]. 

Previously, BAK1-INTERACTING RECEPTOR-LIKE KINASE1 (BIR1) was identified as a negative regulator of plant immunity, as the BIR1 loss-of-function mutant *bir1-1* caused the constitutive activation of cell death and pathogen defense responses [78]. However, recent studies from the same group indicated that the activation of cell death and defense responses in *bir1-1* requires BAK1 kinase activity, indicating that BAK1 functioned as a positive regulator in the BIR1-mediated cell death pathway [79]. This finding is in contrast with the result from the *bak1-4 bkk1-1* mutant, where the autoimmune phenotype or constitutive cell death response of *bak1-4 bkk1-1* was caused by the simultaneous knockout of BAK1 and its close homologue BKK1 (SERK4), suggesting that BAK1 is a negative regulator of the cell death process [80]. Therefore, BAK1 can positively or negatively regulate plant immunity and cell death responses. Recently, the level of BAK1 protein was found to be critical to the control of cell death, as both down- and upregulation of BAK1 could result in spontaneous cell death [81]. In the *bak1-4 bik1* mutant, constitutive cell death was mainly observed in the emerging young leaves, and a much higher BAK1 protein accumulation was detected in emerging leaves than in older leaves [82]. 

### 4.2. Crosstalk at the Cytoplasmic Level

In plant immunity, upon the perception of PAMPs by respective receptor kinases, the complex will activate the downstream MAPK signaling [8,83,84,85]. The MAPKs (mitogen-activated protein kinases), comprising MAPKs, MAPK kinases (MKKs), and MAPK kinase kinases (MAPKKKs), form signaling cascades to regulate diverse developmental processes as well as immune responses [85,86,87]. Studies have shown that at least six members of the MAPKs, MPK1, MPK3, MPK4, MPK6, MPK11, and MPK13, are activated upon flg22 treatment [88,89,90,91,92]. In a recent study in *Arabidopsis*, MAPKKK3/5, MKK4/5, and MPK3/6 were found to form a signaling cascade, transducing defense signals downstream of multiple plant receptor kinases and regulating PAMP-triggered plant immunity. Loss of MAPKKK3/5 leads to compromised MAPK activation and increased susceptibility towards pathogen attack [85]. Recently, the RLCK BSK1 was reported to regulate plant immunity by phosphorylating MAPK kinase kinase 5 (MAPKKK5), which suggested a direct regulatory mode of signaling from the immune complex to the downstream MAPK cascade [93]. 

BIN2 is a GSK3/Shaggy-like protein kinase and negatively regulates the BR signaling pathway [94]. It was previously reported that BIN2 promotes stomata production by phosphorylating and inactivating the MAPKKK YODA (YDA) in the YDA–MKK4/5–MPK3/6 pathway. BR stimulates the YDA–MKK4/5–MPK3/6 pathway to inhibit stomata production through inhibiting BIN2 activity [93,95]. In the recent study, Sun et al. [85] showed that loss of YDA or blocking of BR signaling led to increased PAMP-induced activation of MPK3/MPK6. Moreover, the BIN2 gain-of-function mutant *bin2-1* had stronger flg22-induced MAPK activation. Thus, the authors proposed that BIN2 phosphorylates and inactivates YDA, thereby shifting the MAPK cascade to the formation of the MAPKKK3/5–MKK4/5–MPK3/6 cascade and achieving pathogen defense. Conversely, BR inhibits defense signaling by promoting the formation of the YDA–MKK4/MKK5–MPK3/MPK6 cascade, which would compete for the limiting MKKs with the MAPKKK3/5–MKK4/5–MPK3/6 pathway [85]. These results revealed an antagonistic interaction between a developmental MAPK pathway and an immune-signaling MAPK pathway. 

### 4.3. Crosstalk at the Transcriptional Level

The crosstalk between BR signaling and plant immunity also occurs at the transcriptional level. BZR1 and BES1 are two homologous transcription factors that positively regulate BR signaling and plant growth, and they control thousands of BR-regulated genes [96,97]. BZR1 was recently shown to mediate the trade-off between plant innate immunity and growth [98,99]. On one hand, BZR1-mediated BR signaling promotes the expression of transcription factor HBI1, a positive regulator of BR synthesis and BR-regulated growth, but a negative regulator of immunity [99]. HBI1 was shown to negatively regulate a subset of genes involved in immunity and inhibit PTI-induced growth arrest and a series of defense responses. On the other hand, BZR1 promotes the expression of WRKY transcription factors that negatively regulate immunity (such as WRKY11, WRKY15, and WRKY18) and repress the expression of immune genes by interacting with WRKY40 [13,98]. These studies establish a mechanism by which BZR1, as a BR-activated central growth regulator, directly regulates the expression of defense-related genes; as such, it functions as an integration node of growth and defense pathways to mediate the growth and immunity trade-off in plants.

In contrast to BZR1, BES1 has been shown to increase plant resistance to bacterial pathogens, but enhance susceptibility to necrotrophic fungal pathogens [96]. BES1 was previously reported to target the transcription factor MYB30 to positively regulate the hypersensitive cell death program in plants in response to bacterial and fungal pathogens [100], likely through modulating the expression of defense-related genes. Recently, it was found that BES1 is a direct substrate of MPK6 in PTI signaling and PAMP perception enhances phosphorylation of BES1, resulting in enhanced resistance to the bacterial pathogen *Pseudomonas syringae* pv *tomato* DC3000 [101]. However, BES1 seems to negatively regulate resistance to necrotrophic fungal pathogens, as the *bes1-D* gain-of-function mutant showed higher susceptibility to the necrotrophic fungus *Alternaria brassicicola* [60]. BES1 may participate in the JA-associated defense response towards necrotrophic pathogens [60]. These studies also imply that BZR1 and BES1, as closely related homologous proteins, function similarly in growth and development regulation, but act differently in plant defense and immunity.

## 5. Conclusions and Future Perspectives

Significant progress has been made in the past few years in understanding the mechanisms of how BR regulates plant–microbe interactions, summarized as follows: (1) BR impacts plant interactions with all three types of pathogens (biotrophs, hemibiotrophs, and necrotrophs), but their effects (inducing either resistance or susceptibility) are highly dependent on the trophic types of the pathogens as well as the plant species involved. BR seems to induce resistance to most biotrophic pathogens, but susceptibility to most hemibiotrophic and necrotrophic ones (Figure 2). (2) BR interacts with PTI signaling at multiple levels (receptor, cytoplasmic, and transcriptional), but the interaction outputs seem to be unidirectional: the activation of BR signaling inhibits PTI responses, but the activation of PTI has little effect on BR signaling outputs. (3) BR inhibits plant immune (PTI) responses through both BAK1-dependent and -independent mechanisms. (4) BR has emerged an important regulator of growth–immunity trade-off and several BR signaling components (BRI1, BAK1, BIN2, BZR1, and HBI1) appear to be involved (Figure 1). Despite these exciting developments, there are still some outstanding questions to be addressed in the future. 

Firstly, if BR negatively affects PTI responses, then how does BR induce different plant responses to different pathogens, especially in the same trophic category? Is it determined by the PAMP–PRR specificity of individual pathogens and thus triggers different local and/or systemic defense responses, or is it due to the interplay effects of different hormones, or even due to different experimental conditions used by different researchers? Are there any other mechanisms involved? All these questions need to be clarified by more future studies. Secondly, the finding of a negative and unidirectional interaction between BR–PTI signaling is interesting, but also intriguing. Given the multilevel interactions between the two pathways, the alteration of PTI signaling is expected to have an impact on BR signaling as well. In fact, a recent study indicated that the activation of PTI by the bacterial PAMP flg22 resulted in reduced expression of BR biosynthetic genes, and this effect did not require BR perception or signaling, suggesting that the crosstalk between PTI and BR could actually be negative and bidirectional [14]. In the future, efforts should be made to investigate whether the activation of PTI can affect other BR-regulated processes. Thirdly, it is clear that BR participates in the regulation of the trade-offs between growth and defense; however, several other hormones including GA, ethylene, JA, and SA are also involved. How these different hormones interact to ensure specificity and plasticity in response to different environmental conditions remains to be an important question to answer. Finally, the plant defense system comprises several different mechanisms, including morphological or physical barriers, chemical defense, and the innate immunity. Current research is overwhelmingly focused on how BRs impact plant immunity pathways, and little information is available regarding how BRs modulate the other two mechanisms. It has been previously reported that approximately 10% of BES1 target genes are related to biotic stress responses [40], but currently, only a few of them are assigned with a function to plant immune responses through genetic functional studies. Understanding the functions of all these BES1 target genes (and also of BZR1 targets) in combination with other mechanistic studies will shed more light on the complete mechanisms of BRs in regulating plant defense.
